# Comparative Clinical Behavior of Zirconia versus Titanium Dental Implants: A Systematic Review and Meta-Analysis of Randomized Controlled Trials

**DOI:** 10.3390/jcm13154488

**Published:** 2024-07-31

**Authors:** Danilo Morena, Bruno Leitão-Almeida, Miguel Pereira, Rodrigo Resende, Juliana Campos Hasse Fernandes, Gustavo Vicentis Oliveira Fernandes, Tiago Borges

**Affiliations:** 1 Faculty of Dental Medicine, Universidade Católica Portuguesa, 3504-505 Viseu, Portugal; 2 Centre for Interdisciplinary Research in Health (CIIS), Universidade Católica Portuguesa, 3504-505 Viseu, Portugal; 3 Faculty of Dentistry, Fluminense Federal University, Niterói, RJ 24220-140, Brazil; 4 Independent Researcher, St. Louis, MO 63104, USA; 5 Missouri School of Dentistry and Oral Health, A. T. Still University, St. Louis, MO 63104, USA

**Keywords:** dental implants, zirconia implants, zirconium, titanium implants, titanium, zirconia oxide, yttria-stabilized tetragonal zirconia

## Abstract

**Objective:** The goal of this systematic review and meta-analysis was to assess whether there were clinically relevant differences in the treatment of edentulous areas comparing zirconia (Zr) and titanium (Ti) dental implants. The null hypothesis is that no differences can be observed in terms of the clinical parameters; the positive hypothesis I is that Zr implants have generally better results compared to Ti implants; and the positive hypothesis II is that Ti implants have a generally superior result than Zr implants. **Methods:** This review work was registered on the PROSPERO platform, and its development was conducted in accordance with the PRISMA (Preferred Reporting Items for Systematic Reviews and Meta-Analyses) statement. The electronic search process was conducted on three databases (PubMed/Scopus/Web of Science), including randomized controlled trials (RCTs) from the past 10 years (up to April 2024). Identified articles were analyzed and included/excluded based on pre-defined selection and exclusion criteria. The quality assessment and risk of bias were evaluated using a Cochrane risk-of-bias assessment tool specifically designed for randomized trials (RoB2). A meta-analysis was conducted to correlate different treatment options based on the described outcomes; a random-effects model was used in the analysis of the variables. The analysis of heterogeneity was conducted by means of Cochran’s Q-test and Higgins’ I^2^ statistic. **Results:** Six RCTs were enrolled; 152 patients (90 males and 62 females) and 448 implants (267 Zr and 181 Ti) were included. Dental implant placement involved both the maxillary and mandibular arches. The implant sites showed heterogeneity in receiving Zr and Ti dental implants; in particular, 22 dental implants were placed in the mid-palatal region and 426 dental implants in the alveolar region (255 were in Zr and 171 in Ti). Regarding the success rate, it was better for Zr but with no statistical difference (*p* > 0.05); bleeding on probing had slight differences between Ti with 0.34% ± 0.42 and Zr with 0.26% ± 0.36 (*p* > 0.05); plaque score showed 0.46 ± 0.47 for Ti compared to 0.44 ± 0.49 for Zr (*p* > 0.05); no statistically significant difference was observed for pink esthetic score (PES). Statistically significant results were found for survival rate, which favored Ti implants (77.6%) compared to Zr (70.3%) (*p* < 0.05), and for marginal bone loss, which showed less loss in Ti implants (0.18 mm ± 0.47) compared to 0.42 mm ± 0.40 in Zr at 12 months (*p* < 0.001). **Conclusions:** The present systematic review and meta-analysis identified the positive hypothesis I and rejected the null and positive hypothesis II; it was possible to conclude that Ti dental implants have a better survival rate and less marginal bone loss than Zr dental implants after 1-year follow-up.

## 1. Introduction

Edentulism has been illustrated as an oral handicap that presents functional, aesthetic, and psychosocial limitations in affected patients [1]. Despite the refinement of techniques to provide retention and stability to mucosa-supported prostheses, there are limitations, especially in the mandibular bone region, due to the reduced functional surface area and tongue movements, which cause the prosthesis to tilt. In addition, they can accelerate the resorption process of the alveolar bone, given the uneven underlying pressure exerted. There is a high number of reports of patients expressing dissatisfaction with masticatory and phonetic function, who never fully adapt and continue to perceive these rehabilitations as foreign bodies [2].

The decisive breakthrough came in 1965 when Dr. Branemark placed the first dental implant in titanium (Ti), finally providing an alternative to the various rehabilitation protocols with promising solutions in terms of functionality [3]. To achieve a good result from a functional point of view, it is essential to establish osseointegration through direct contact between bone and implant without the presence of fibrotic tissue. From a biological point of view, dental implants are tolerant to the peri-implant region, being recognized by the body as a foreign body, causing a defense reaction with the formation of new bone around the implant. From a clinical point of view, the term osseointegration is considered to implant stability and osseointegration [4]. Some clinical parameters are important sources of information for dental implants. One of them was mentioned by Albrektsson and Isidor, who reported a marginal bone loss (MBL) of up to 1.5 mm in the first year and around 0.2 mm in subsequent years as a factor in dental implant success. This parameter may be influenced by clinical, mechanical, and biological factors [5], such as implant design, crestal width, overheating of the bone, surgical trauma, and functional occlusal overload [6].

Ti dental implants present high biocompatibility, a success rate in various ten-year follow-ups of 95% [6,7], and a survival rate of 96.4% [4]. However, Ti has been shown to become corrosive in environments with low pH and oxygen levels, releasing Ti ions when subjected to mechanical stress [1]. The immune system reacts to the presence of free Ti particles around implants, which are by-products of corrosion [8], being phagocytized by activated macrophages and neutrophils as part of the biological response [9]. The body’s response can take the form of type IV hypersensitivity reactions [6]. This results in signs such as erythema, urticaria, eczema, swelling, pain, necrosis, and bone loss, with the potential risk of implant failure [10,11]. Furthermore, the use of Ti implants in the esthetic area may entail the risk of discoloration of peri-implant soft tissue, especially in the presence of localized bone loss or a thin gingival biotype [1,7], resulting in the observation of greyish peri-implant soft tissue [11].

Ceramic materials used as dental implants were first introduced and used clinically in the 1960s [12]. Compared to previously considered materials, dental implants made of zirconia (Zr) have a higher flexural strength, a lower modulus of elasticity, and a higher fracture toughness [12,13], wear and corrosion resistance, as well as high structural strength [6]. They have high biocompatibility and, being metal-free, also exhibit a lower inflammatory response, thus reducing the risk of allergic reactions or irritation [12]. The clinical results of the Zr implants showed a success rate of 98% at the 1-year follow-up [10]. Some structural modifications can bring changes to dental implants, improving, for example, their stiffness and strength. Then, a Ti-Zr alloy emerges as an alternative exhibiting interesting properties, showing fewer inflammatory and tissue processes than Ti. It has greater strength than Ti, thus reducing the risk of fracture. Studies reveal a survival rate of 97% at 3-year follow-up, as well as showing osseointegration similar to that of Ti. In the case of peri-implant bone dehiscence and fenestrations, the literature recommends the use of implants with smaller diameters, reducing the risk of recession or resorption [13,14], and avoiding bone regeneration, simplifying the surgical protocol [2]. This Ti-Zr alloy has achieved high biocompatibility and high strength [12], showing the mechanical properties of Ti, providing the biological benefits of Zr, and maintaining similar results in terms of osseointegration [13,14]. However, it does not address the aesthetic issue, particularly in terms of coloring, similar to that of Ti implants [6].

Ti dental implants are still reputed to be the gold standard implant material, presenting good strength [6,14,15], osseointegration, biocompatibility [3,6,13,16], and a success rate ranging from 98% at 1-year follow-up and 95% at 2.5–5-year follow-up [12]. Zr implants exhibit high biocompatibility with a success rate of 97% after 1 year [11] and 93% afterward in a follow-up of 2–3 years [12], proving to be a viable alternative in the case of Ti allergy [3,4]. Other advantages associated with Zr implants are the absence of adverse reactions such as hypersensitivity responses, as well as having a coloring similar to natural teeth [3,4,12]. They also exhibit flexural strength, radiopacity [4,12], plaque control [8,9] toughness, as well as good osseointegration [3,4]. In addition, Ti-Zr alloy implants (85% Ti-15% Zr) have demonstrated biocompatibility (a better response to cell proliferation than classical Ti alloys [13,14]) and the strength of Zr and Ti and a favorable elasticity modulus [3].

Thus, the goal of this systematic review and meta-analysis was to assess whether there were clinically relevant differences in the treatment of edentulous areas comparing Zr dental implants and the established use of Ti dental implants. The null hypothesis is that no one difference can be clinically observed; the positive hypothesis I is that Zr implants have generally better results compared to Ti implants; and the positive hypothesis II is that Ti implants have a generally superior result than Zr implants.

## 2. Materials and Methods

The protocol followed adhered to the PRISMA (Preferred Reporting Items for Systematic Reviews and Meta-Analyses) statement [17]. This systematic review has been registered on PROSPERO (CRD42024489015).

### 2.1. PICO Question

The PICO (Population, Intervention, Comparison, and Outcome) strategy was used as a template to formulate the clinical question. The focused question was as follows: “In patients in need of one or more dental implants (P), are there any clinically relevant differences (O) between the treatment of edentulous areas with Zirconium implants (I) compared to the use of Titanium implants (C)?”.

The primary outcomes observed were the implant survival rate, implant marginal bone loss (MBL), and implant success rate. The secondary endpoints/outcomes were as follows: bleeding on probing (BoP), plaque index (PI), pink esthetic score (PES), and reasons for failure.

### 2.2. Eligibility Criteria

The inclusion criteria for this review were as follows: (1) adult patient (≥18 years old), (2) randomized clinical trials (RCTs), (3) follow-up ≥3 months, (4) articles published in the English language (5) in the last 10 years, and (6) focus on dental implant clinical behavior. The exclusion criteria were as follows: (1) animal studies, (2) cohort studies, (3) clinical controlled trials, (4) randomized clinical trials older than 10 years, (5) in vitro studies, (6) case reports, (7) case series, (8) any RCTs that included patients with non-controlled systemic disease, and (9) focus on abutments’ results.

### 2.3. Source of Information and Search Strategy

Two independent authors (T.B. and D.M.) conducted the electronic search on three databases: Medline/PubMed, Scopus, and Web of Science (WoS). In case of disagreement, a tie-break was performed by a third author (G.V.O.F.). The PICO question guided the search parameters, and search filters were applied. These filters included a temporal restriction to the past ten years, articles composed in English, randomized controlled trial (RCT) studies, and exclusively clinical and human studies involving adult patients (Table 1). The inter-reliability level of agreement was measured through Cohen’s kappa test.

### 2.4. Quality Assessment, Risk of Bias, and Meta-Analysis

The quality of the conducted study was independently assessed by two authors (T.B. and D.M.). In the case of disagreement, a third author was consulted (G.V.O.F.). The risk of bias in the considered RCTs was evaluated using a Cochrane risk-of-bias assessment tool specifically designed for randomized trials (RoB2). The domains of the tool (RoB2) included the randomization process, deviations from the intended interventions, missing outcome data, outcome measurement, and selection of reported outcomes. The options to qualify each item were as follows: (1) low risk; (2) some concern; and (3) high risk. In the case of ≥2 “high risk”, the overall result will be “high risk”; with up to 2 “some concern” present, a “low risk” will be found in the overall result.

All statistical analyses were conducted using Review Manager 5.4 (https://revman.cochrane.org/). For variables where meta-analysis was not applicable, a comparative analysis of study results was employed. The random-effects model was used in the analysis of the variables. The analysis of heterogeneity is conducted by means of Cochran’s Q-test and Higgins’ I^2^ statistic. Standardized mean differences were used as a method for measuring the effect. By dividing the mean difference of each study by its standard deviation, a comparable measure between the studies is obtained.

## 3. Results

### 3.1. Study Selection

**Figure 1:**
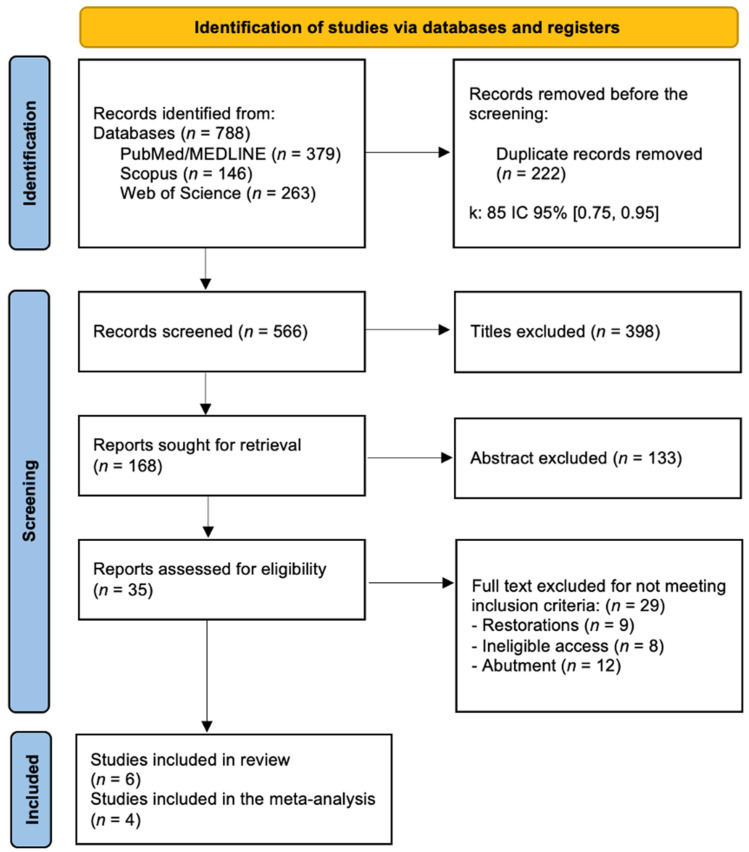
Flow diagram describing the selection process according to PRISMA (Preferred Reporting Items for Systematic Reviews and Meta-Analyses).

The PRISMA diagram was used to show the workflow (Figure 1). The electronic search yielded 788 articles (PubMed, Scopus, and WoS). A total of 222 duplicates were removed, leaving 566 articles. After analyzing the titles, 398 publications were excluded, leaving 168 articles. After an evaluation of the abstracts, 133 were removed. Of the remaining 35 eligible articles, 29 were excluded for reasons such as a greater focus on the duration of restoration rather than the implants and on the abutment instead of on the dental implant. Six articles were included in the study that met the predetermined inclusion criteria. Of these six, only four presented comparable results through a meta-analysis (k = 0.93).

### 3.2. Study Characteristics

All studies included compared the use of Ti and Zr dental implants at follow-up, addressing the selected criteria. Extrapolated data on gender revealed the presence of 90 males and 62 females (total *n* = 152) who underwent implant surgery [1,8,9,11,12,18]. In Table 2, we can see the demographic information on the number of patients, gender, and average age. In some articles, the group was specified, whether Ti or Zr. Dental implant placement involved both the maxillary and mandibular arches. The implant sites showed heterogeneity in receiving Zr and Ti dental implants. In particular, 22 dental implants were placed in the mid-palatal region and 426 dental implants in the alveolar region, of which 255 were in Zr and 171 in Ti (Table 2). In Table 2, it is possible to see the type and number of dental implants, their position in the oral cavity where they were placed, the surgical protocol used, final/interval follow-up, and reported outcomes. In Table 3 and Table 4, the study description and results for the predetermined outcomes are presented. Supplementary Table S1 presents the outcomes in an integral manner, presenting the results of the studies in more detail.

### 3.3. Study Characteristics

The implant sites showed heterogeneity in receiving Zr or Ti dental implants. Four articles provided dental implant placement in the anterior/posterior region of the maxilla and mandible (*n* = 341), of which 183 were in Zr and 158 in Ti [1,9,12,18]. One article considered insertion only in the anterior area of the jaw (*n* = 30), of which 16 were in Zr and 14 in Ti [11]. Another article inserted implants in the posterior region of the maxilla and mandible (*n* = 80), of which 40 were for Zr and Ti, respectively [8] (Table 2).

Three studies showed smoking patients as an exclusion criterion [1,9,18]. Two studies showed heavy smoking patients (more than 10 cigarettes/day) as an exclusion criterion [11,12]. One study identified heavy smokers (more than 15 cigarettes/day) as an exclusion criterion [8]. Regarding the periodontal status, active periodontal disease has been listed among the exclusion criteria [9,11,18]. Regarding the condition of the alveolar bone, all articles, except Henao et al. [11], highlighted sufficient quantity and quality of bone among their inclusion criteria.

### 3.4. Single-Piece Zr Implants vs. Ti Implants for Supporting Overdentures

Siddiqi et al. [1] and Osman et al. [12] compared the use of Zr implants with Ti implants for overdenture support. Regarding the survival rate, the results show in favor of Ti implants (77.6%) compared to Zr (70.3%). Regarding the MBL parameter, it can be stated that the results show less marginal bone loss in Ti implants, with a loss of 0.18 mm (SD 0.47) compared to 0.42 mm (SD 0.40) in Zr. Regarding the BoP parameter, Siddiqi et al. [1] showed slight differences between Ti with 0.34% (SD 0.42) and Zr with 0.26% (SD 0.36). The plaque score showed 0.46 (SD 0.47) for Ti compared to 0.44 (SD 0.49) for Zr. The success rate was better for Zr, with a result of 67.6% Zr compared to 66.7% Ti for Siddiqi et al. [1] and 57.5% for Zr compared to 57.1% for Ti [12].

### 3.5. Single Dental Implants in Ti versus Zr Single-Piece in the Anterior Maxilla

Henao et al. [11] showed a higher survival and success rate for Ti (100%) than Zr (97%). In relation to the MBL parameter, a marginal bone loss for Ti implants of 0.16 mm (SD 0.32) compared to 0.28 mm (SD 0.27) for Zr was observed. The BoP showed a result of 0.05% (SD 0.10) for Ti compared to 0.26% (SD 0.42) for Zr. The plaque index (PI) parameter showed a value of 0.01 (0.06) for both Zr and Ti groups. Regarding the PES parameter, Ti implants reported a score of 7.86 (SD 1.29) compared to 7.81 (SD 1.72) for Zr.

### 3.6. Full Ceramic Restoration of Two-Piece Zr Implants vs. Ti Implants

Payer et al. [18] and Koller et al. [9] predicted the placement of two-piece Zr implants. The results for the MBL parameter showed a result for Zr of 1.16 mm (±1.01; SE 0.8), compared to 0.88 mm (±0.56; SE 0.88) for Ti according to Payer et al. [16], whereas Koller et al. [9] showed a result of 1.51 mm (SD 0.68; ME1.48) for Zr, compared to 0.92 mm (SD 0.72; ME 1.03) for Ti at 80 months of follow-up. The BoP score was 11.9% (±9.44; SE 9.5) for Zr implants, while for Ti implants, the score was 7.9% (SD ± 4.98; ME 7.9) according to Payer et al. [16] compared to 10.05% (SD 6.43; ME 10.5) for Zr versus 15.46% (SD 5.67; ME 16) for Ti according to Koller et al. [9].

Considering the PI parameter, it showed a result of 15.88% (SD 6.67; ME 12.0) for Zr versus 11.19% (SD 5.69; ME 9.8) for Ti according to Payer et al. (2015), while Koller et al. showed a result of 11.38 (SD 0.92; ME 11) for Zr versus 11.14 (SD 1.07; ME 11) for Ti. Considering the PES, a value of 10.33 (±2.06; SE 9) was observed for Zr implants, compared to 9.0 (±3.54; SE 10) for Ti implants according to Payer et al. [16] and 1.51 (SD 0.68; ME1.48) for the Zr group, compared to 0.92 (SD 0.72; ME 1.03) for the Ti group according to Koller et al. [9] at 80 months. The survival rate was higher in the Ti group (93.3%) than in the Zr group (85.7%), according to Koller et al. [9].

### 3.7. One-Piece Zr Implants versus Ti Implants under Healthy and Experimental Mucositis Conditions

Bienz et al. [8] placed more emphasis on the BoP parameter, which showed a mean of 11.67 (SD 18.81) for the Zr group, whereas a mean of 11.97 (SD 16.31) was recorded for the Ti group, showing a better bleeding index at probing under mucosal conditions for Zr. Table 3 shows the results for the predetermined outcomes. Supplementary Table S1 has more detailed information on the outcomes.

### 3.8. Risk of Bias

**Figure 2:**
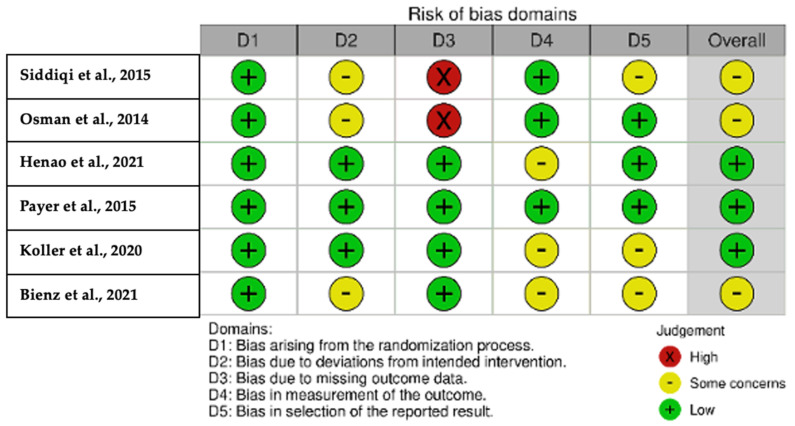
Overall assessment of bias risk using the Cochrane Risk of Bias 2 (RoB 2) tool [1,8,9,11,12,16].

**Figure 3:**
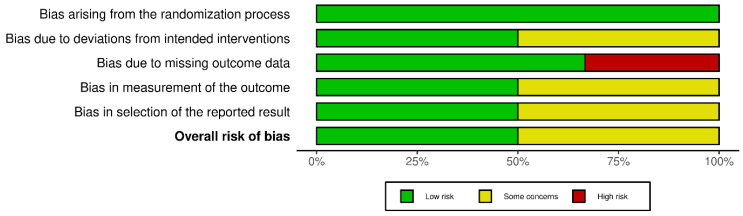
Overall risk-of-bias assessment using the Cochrane Risk of Bias 2 (RoB 2) tool.

A high risk of bias was found in two studies (one parameter) [1,12], whereas a moderate risk was found in the overall result of three articles [1,8,12]. Low risk was detected in three studies [9,11,18] (Figure 2 and Figure 3).

### 3.9. Meta-Analysis and Clinical Outcomes

A meta-analysis of four randomized clinical trials was conducted, analyzing the results at 1-year follow-up. Implant surgery was performed on 90 patients, with the placement of 420 implants. Specifically, 265 implants belonged to the Zr dental implant, while 165 belonged to the Ti dental implant. Table 5 shows the number of implants of each type analyzed in each study. It was concluded that the majority of implants in all studies were of type Zr, but this difference was not statistically significant.

**Figure 4:**
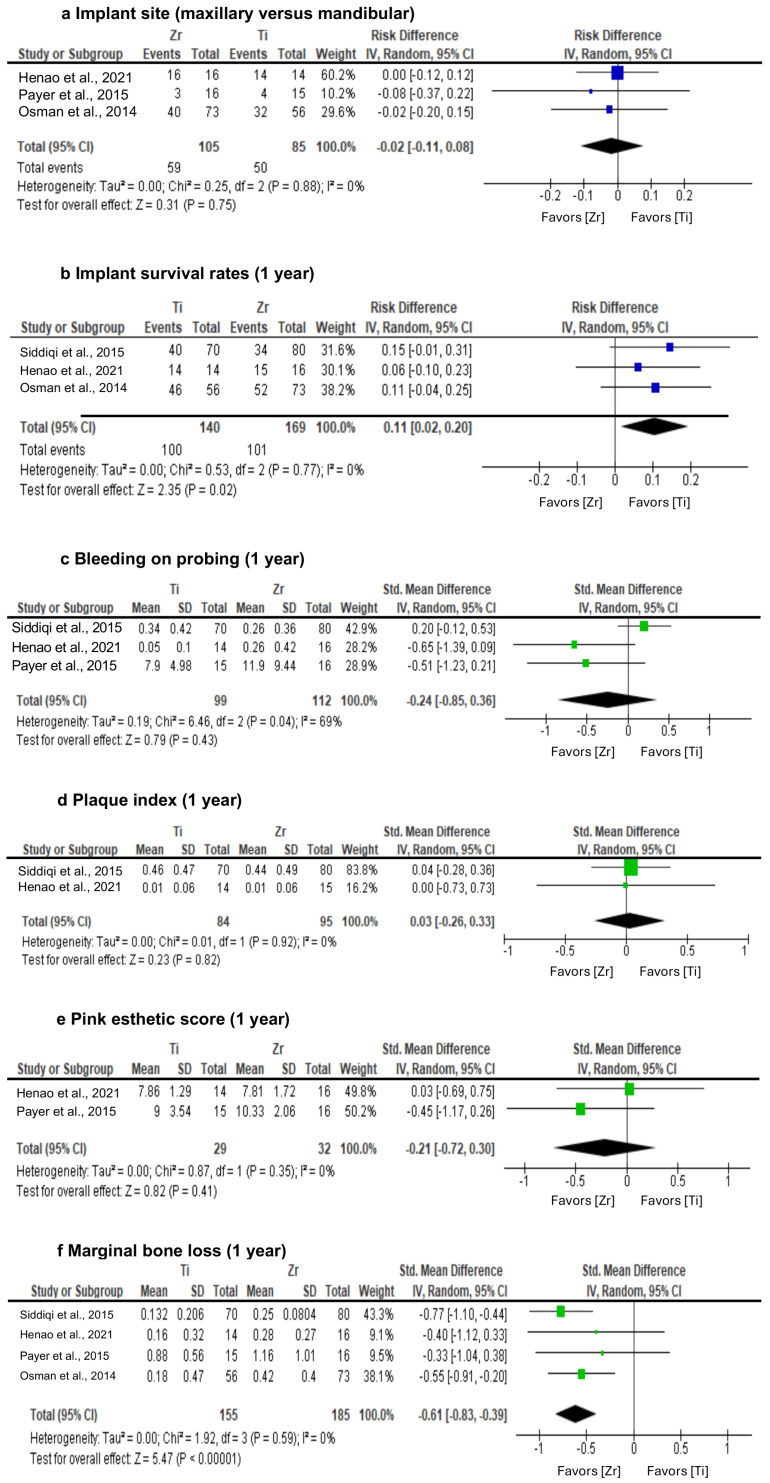
Results of meta-analysis for: (**a**) implant site (maxillary versus mandibular); (**b**) implant survival rates (1 year); (**c**) bleeding on probing (1 year); (**d**) plaque index (1 year); (**e**) pink esthetic score (1 year); and (**f**) marginal bone loss (1 year) [1,11,12,18].

For the implant site, observing the forest plot, it is noted that in the studies analyzed, the majority of maxillary implants occurred with Ti implants; the effect of the meta-analysis is not statistically significant (*p* = 0.75) (Figure 4a). Based on the results for the implant site, Cochran’s Q (*p* = 0.88) and I^2^ = 0%, there was homogeneity among studies.

For implant survival rate, when analyzing the results of each article included in this review, it could be seen that Ti implants have a higher survival rate according to the various studies taken into consideration [1,9,11,12,18]. Observing the forest plot presented, Ti implants had the highest percentage of successful implants after one year, and the effect of the meta-analysis was 0.11 (95% CI [0.02; 0.20]), statistically significant (*p* < 0.05). Thus, it can be stated that the success of Ti implants after one year was statistically significant compared to Zr implants in the analyzed studies. Based on the results, Cochran’s Q (*p* = 0.77) and I^2^ = 0%, there was homogeneity among studies for this parameter (Figure 4b).

The success rate, according to Siddiqi et al. [1] and Osman et al. [12], was slightly higher for Zr implants (67.6% Zr, compared to 66.7% Ti, and 57.5% Zr compared to 57.1% Ti). Henao et al. [11] found 97% for Zr compared to 100% for Ti. Two studies did not mention this parameter.

For BoP, Siddiqi et al. [1] and Koller et al. [9] revealed favorable BoP results in Zr implants. Henao et al. [11], Payer et al. [18], and Bienz et al. [8] revealed a favorable BoP parameter for Ti implants. Henao et al. [11], with 0.26% (SD 0.42) for Zr implants, was the best score obtained. Only one article did not consider the evaluation of the BoP parameter [12]. It was possible to note in the forest plot that the effect of the meta-analysis was −0.24 (95% CI [−0.85; 0.36]), meaning a not statistically significant result (*p* = 0.85) (Figure 4c). Based on the results, Cochran’s Q (*p* < 0.05) and I^2^ = 69%, it was concluded that there was significant heterogeneity among studies regarding this parameter (BoP). This heterogeneity can be justified by Payer et al.’s results [18], which were an order of magnitude higher than the other studies.

For plaque index, Siddiqi et al. [1] showed favorable plaque index results for implants in Zr. Payer et al. [16] and Koller et al. [9] showed favorable plaque control in Ti. Henao et al. [11] presented the best plaque result for both groups: 0.01 (SD 0.06). Two articles did not consider the plaque index in their results [9,12]. Observing the forest plot, it is noted that the effect of the meta-analysis was 0.03 (95% CI [−0.26; 0.33]), which was not statistically significant (*p* = 0.82). Based on the results, Cochran’s Q (*p* = 0.15) and I^2^ = 0%, there was no heterogeneity among studies after 1 year (Figure 4d).

For the pink esthetic score (PES), Henao et al. [11] showed a better result for the use of Ti implants. Payer et al. [18] and Koller et al. [9] showed a favorable PES value for Zr dental implants. The best result was obtained by Koller et al. [9] in restoration with two-piece Zr implants, showing a score of 10.33 (SD 2.06; ME 9) for Zr compared to 9.0 (SD 3.54; ME 10) for Ti. Three articles did not consider the PES as an outcome variable [1,8,12]. The forest plot shows that the effect of the meta-analysis was −0.21 (95% CI [−0.72; 0.30]), which was not statistically significant (*p* = 0.41). Based on the results of this parameter, Cochran’s Q (*p* = 0.35) and I^2^ = 0%, there was no significant heterogeneity among studies.

With regard to the parameter marginal bone loss (MBL), five articles showed a better result for Ti [1,9,11,12,18]. The effect of the meta-analysis was −0.61 (95% CI [−0.83; −0.39]), which was statistically significant (*p* < 0.001), which allows us to state that the results obtained for MBL were significantly lower in Ti implants compared to Zr implants in the analyzed studies. Based on the results, Cochran’s Q (*p* = 0.59) and I^2^ = 0%, it was concluded that there was no heterogeneity among the studies for this parameter.

## 4. Discussion

Thus, the goal of this systematic review was to assess whether there were clinically relevant differences in the treatment of edentulous areas comparing Zr dental implants and the established use of Ti dental implants. Therefore, the number of randomized controlled clinical studies comparing the use of these two materials (Ti and Zr) is relatively small, and the great part of the scientific evidence in the literature on Zr dental implants mainly concerns in vitro studies or in animal models [1,4], which showed interesting results.

Regarding Ti and Zr supporting rehabilitation, specifically overdenture, Siddiqi et al. [1] and Osman et al. [12] showed results on the comparison between Zr and Ti implants as overdenture support. Siddiqi et al. [1] presented a higher survival rate for Ti implants than for Zr implants, 77.6% and 70.3%, respectively, at 1-year follow-up, whereas Osman et al. [12] presented 71.2% for Zr dental implants versus 82.1% for Ti implants, applying the same implant rehabilitation procedure. The comparative statistical results between the two studies showed a difference of 0.15 (−0.01; 0.31) for Siddiqi et al. [1] compared to 0.11 (−0.04, 0.25) for Osman et al., [12] in favor of Ti implants. Concerning rehabilitation with two-piece Zr implants for single-area restorations or up to 3 units, Koller et al. [9] showed a survival rate of 85.7% for Zr versus 93.3% for Ti in a 30-month follow-up. Henao et al. [11] investigated the placement of one-piece dental implants for single-unit restorations in the anterior maxillary area; the authors showed lower results for Zr implants, with a survival rate of 97% versus 100% for Ti at 1-year follow-up. These findings are in line with studies by Kohal et al. [19], who showed a survival rate of 95.4% for one-piece Zr; Mellinghoff et al. [20], with 93% for Zr dental implants at 1-year follow-up; Oliva et al. [21], with 97.4%; and Duan et al. [17], with 92.77% at 1-year follow-up.

For survival rate, when analyzing a follow-up period of 80 months, it was found that the Ti survival rate continued showing better results than Zr [9]. Ti-Zr alloy has higher survival and success rates than Zr implants, and similar to Ti implants [2,6,16]. A follow-up showed a survival rate for the Zr implants of 91% and 95.8% for Ti implants, whereas, for Ti-Zr, it was 100% [3]. Our meta-analysis shows Ti as having a better survival rate than Zr, confirming results from the literature [1,9,11,12,18] and showing a result with a statistically significant effect of 0.11 (95% CI [0.02; 0.20] at 1-year follow-up (*p* < 0.05). Among the included studies in this systematic review, Henao et al. [11] had the lowest statistical difference score between Ti and Zr implants in single-unit anterior maxillary bone rehabilitation treatment. Osman et al. [12] demonstrated a different response of the maxillary bone (55%) compared to the mandibular bone (71.9%). In agreement with previous studies, a higher failure rate for implants placed in the maxilla was noted, either in Zr or Ti [1,12]. Moreover, regarding the jaw, the authors of [12] agreed with Lambrich and Iglhaut’s study [22], presenting a survival rate of 98.4% in the mandible and 84.4% in the maxilla.

A systematic review evaluated the histological osseointegration between Ti and Zr [4]. The osseointegration rates achieved similar levels (%bone–implant contact [BIC]), with an average BIC for Zr of 55.51% and for Ti of 58.50%, which increased with time and reached similar values. Comparing Zr and Ti one and two pieces, a similar osseointegration rate was reported.

Another systematic study [7] reported that the majority of the zirconia implants evaluated in their study were a single-piece design that represents a supramucosal part (abutment), which was reported to overcome bacterial accumulation and consequent crestal bone resorption, different from two-piece implants which can have the presence of a microgap due to the interface between the abutment and its implant platform, possibly compromising the MBL. The literature supports that more inflammation is found in titanium implants, which can be represented initially by the BoP and, consequently, MBL [23]. This fact was proved by studies using zirconia implants, showing reduced activation of local inflammatory response and bone resorption compared with titanium [24,25].

Moreover, this same review [7] reported negative aspects found for Zr implants. Implant fractures were found, totaling 22 Zi implants, which represented 1.7% of the total number studied (between 0.8 and 69.7 months (mean period of 15.3 months) after implant placement), and a great part of them, almost 70%, involved narrow implants (3.25 mm in diameter) and were placed in the maxilla; otherwise, none of the implants with 5.0 mm diameter fractured.

### 4.1. Clinical Parameters

#### 4.1.1. BoP and Plaque

When analyzing the BoP results in the different studies, Siddiqi et al. [1] showed a result of 0.26 (±0.36) for Zr compared to 0.34 (±0.42) for Ti. The statistical difference was 0.20 [−0.12; 0.53], favoring the Zr group. Henao et al. [11] showed a result of 0.26% (±0.42) for Zr compared to 0.05% (±0.10) for Ti. Payer et al. [18] showed, in their study, a mean statistical difference of −0.51 (−1.23; 0.21) for BoP. The meta-analysis in our study resulted in −0.24 (95% CI [−0.85; 0.36]) (*p* = 0.85), describing different results depending on the rehabilitation protocol. The literature agrees on the lack of significant differences in rehabilitation with Zr or Ti implants [6,9,12] for this parameter.

Under mucositis conditions, a further detachment between the two materials could be observed, with an increased BoP around Ti dental implants compared to Zr implants, 2–3 sites out of 12 around Zr implants, while 4 sites out of 12 for Ti. The Zr implants showed fewer signs of inflammation than the Ti implants [8]. Observing the Ti-Zr implants, they showed better BoP results than the Ti and Zr dental implants [3,6].

Concerning plaque control, Siddiqi et al. [1] presented a score of 0.44 (±0.49) for Zr versus 0.46 (±0.47) for Ti. Henao et al. [11] reported a result of 0.01 (±0.06) for both groups, with no differences in the choice of material used. The meta-analysis performed here showed 0.03 (95% CI [−0.26; 0.33]) for the PI parameter, which was not statistically significant (*p* = 0.82), suggesting a similarity of the two materials for the articles analyzed [1,9,11,16]. However, a slightly better score than Ti is revealed when we consider the use of dental implants in support of overdenture [1], with a statistical difference of 0.04 (95% CI [−0.28; 0.36]) compared to the use of single restorations in the anterior area of the maxilla, where the statistical difference was 0.00 (95% CI [−0.73; 0.73]), showing a total similarity between the two materials.

#### 4.1.2. PES

Analyzing the PES parameter, Henao et al. [11] showed a result of 7.81 (±1.72) in Zr versus 7.86 (±1.29) in Ti. Regarding two-piece Zr dental implants, Payer et al. [16] had a superior PES for Zr, with a score of 10.33 (±2.06; ME 9) versus 9.0 (±3.54; ME 10) for the Ti group. The results are in agreement with a previous study by Kohal et al. [19], suggesting that the implant material may not have a significant impact on the esthetic parameters of peri-implant soft tissue [1]. According to our meta-analysis, −0.21 (95% CI [−0.72; 0.30]), the result was not statistically significant (*p* = 0.41). However, it is important to mention that Henao et al. [11] showed a score of 0.03 (95% CI [−0.69; 0.75]), slightly in favor of Ti implants when compared with the use of one-piece Zr implants. In contrast to this, Payer et al. [18], comparing two-piece Zr dental implants with Ti, showed a difference of −0.45 (95% CI [−1.17; 0.26]) in favor of two-piece Zr dental implants. When choosing this parameter (PES), it should not be overlooked that the most coronal area acquires fundamental importance in the functional and esthetic results of the implant restoration, as it has a thickness limit, below which discoloration of the mucosa manifests itself. Thoma et al. [26] noted that with a thickness of 1.68 mm (±0.91) of the buccal soft tissue, mucosal discoloration is noted for both types of dental implants, being more pronounced with Ti implants given the staining [11]. These differences become particularly relevant in the rehabilitation of esthetic sites with the aggravating factors of localized bone loss or a fine gingival biotype [12].

#### 4.1.3. MBL

Assessing the MBL outcome in the selected studies, it was evident that Ti implants showed less radiographic vertical bone loss in all implant protocols analyzed [1,9,11,12,15,18]. The result of our meta-analysis agrees with the authors’ findings (−0.61 (95% CI [−0.83; −0.39]; *p* < 0.001), confirming that Zr implants showed a higher MBL when compared to Ti implants. Payer et al. [18] and Koller et al. [9] showed no significant differences between Zr and Ti implants in restoring edentulous areas of up to three units. In contrast, Osman et al. [12] and Siddiqi et al. [1] found significant bone loss with a detachment in favor of Ti when using an overdenture; this result is supported by Fernandes et al. [6], who reported lower MBL values for Zr implants than Ti implants, and by Steyer et al., who evaluate Zr implants after 4, 8, and 11 years [27]. The meta-analysis agrees with these results, having shown a statistical difference in the case of implant-supported overdenture rehabilitations of −0.77 (95% CI [−1.10; −0.44]) for Siddiqi et al. [1], and −0.55 (95% CI [−0.91; −0.20]) for Osman et al. [12], which is higher than the result of −0.40 (95% CI [−1.12; −0.33]) obtained by Henao et al. [11] in the case of single-unit rehabilitations in the anterior region of the maxillary bone. It is also important to mention that the two-piece Zr implant solution, proposed by Payer et al. [18], used in implant restorations with up to three dental elements, showed the highest bone loss in the various protocols analyzed (−0.33 (95% CI [−1.04; −0.38]). Two-piece Zr implants ideally present important features by allowing post-insertion modifications facilitated by the possibility of separating the implant from the abutment, offering a greater possibility of customization of the prosthetic superstructure and allowing easier maintenance in the case of replacement of individual parts due to injury or wear [9,18].

However, the interface between the abutment and implant is a possible cause of bacterial accumulation as well as the fact that the assembly protocol is still very complex. Furthermore, for effective adhesion of the abutment on the implant, one of the prerequisites is the application of a rubber dam, which seems to be a challenge [9,18]. One of the disadvantages of this system is the expenditure of time during the visit. Even lower esthetic results may be achieved due to more tissue remodeling caused by the invasiveness required for its application [18]. One of the relevant aspects of this adhesive protocol is the elimination of the microgap between the interfaces [9], which is considered one of the main disadvantages of normal screw connections. One-piece Zr implants will be more challenging for the final restoration. The limitations of the one-piece dental implant result in the impossibility of making changes in the angulation and position of the implant–abutment combination and in the reduced possibility of choice and adaptation of the prosthetic surfaces that follow [9].

The analysis of the results shows that Zr dental implants present favorable results, esthetically and in the analysis of the restoration with peri-implant soft tissue integration [9,18]. The clinical and radiological results were comparable to Ti dental implants, showing itself as a viable treatment option in esthetic areas, with restorations of reduced edentulous areas [9,11,18]. However, there are obvious limitations when Zr dental implants are used in an overdenture, limiting the choice of a Zr implant purely to professional preference or an allergy condition [1,12].

#### 4.1.4. Implant Diameter (ø)

When comparing 3.3 mm Ti-Zr implants with 4.0 mm Ti implants, Ti-Zr implants demonstrated similar values to Ti implants. Reduced-diameter implants have proven to be an effective treatment option for supporting overdentures for fixed prostheses in edentulous patients, being able to withstand high mechanical stresses, such as in the posterior region [13,14]. They showed higher MBL, BoP, and survival rates than Ti and Zr implants. However, they presented a higher incidence of complications, such as screw loosening or abutment fracture [3,6], and there is a need for further studies comparing different treatment options [6]. Further studies are also needed to evaluate Zr implants with long-term follow-up, revealing the suitability of this material as an alternative to Ti, especially in the case of extensive rehabilitations in posterior regions of the mouth or for overdenture support.

### 4.2. Limitations of the Study

The limitations presented within this review are a low number of RCTs available in the literature supporting this topic and the consequently small sample size; the meta-analysis made use of a further reduced number of studies due to the different follow-ups and the consequent impossibility of comparison; due to the different protocols considered in implant restoration, different results were obtained. Because of this, all results must be critically analyzed. Furthermore, the result of the meta-analysis at the 1-year follow-up may not be sufficient, and a long-term follow-up is needed to assess the success of the Ti and Zr dental implants. The quality of the studies was determined through risk assessment, which did not show any result distortions, thereby ensuring that the information is based on solid and impartial evidence.

## 5. Conclusions

Within the limitations of this study and after careful analysis of the results obtained, the positive hypothesis I was confirmed, and the null and positive hypothesis II were rejected; in addition, it was possible to conclude that Ti dental implants have a better survival rate and less marginal bone loss than Zr dental implants after 1-year follow-up. Further RCTs comparing these two different implant materials with a long-term follow-up evaluation are needed.

## Figures and Tables

**Table 1:** The search was conducted using specific keywords per database.

Database	Search Strategy
PubMed/MEDLINE	Dental implants AND Zirconia implants OR zirconium implants OR titanium implants OR zirconium oxide OR Yttria Stabilized Tetragonal Zirconia
Scopus	Dental implants AND Zirconia implants OR zirconium implants OR titanium implants OR zirconium oxide OR Yttria Stabilized Tetragonal Zirconia
Web of Science	(ALL = (Dental implants AND Zirconia implants OR zirconium implants OR titanium implants OR zirconium oxide OR Yttria Stabilized Tetragonal Zirconia)) AND ALL = (Randomized Controlled Trial)

**Table 2:** Demographic information of the selected studies.

Author	Study Design	Patients Number	Age Range	Gender (Male; Female)
Siddiqi et al., 2015 [1]	RCT	24	50–79	15 M; 4 F
		Follow-up: 19	Mean age: 62	73.7%; 26.3%
		Group I (Zr): 11	Group I (Zr): N/D	Group I (Zr): N/D
		Group II (Ti): 8	Group II (Ti): N/D	Group II (Ti): N/D
Osman et al., 2014 [12]	RCT	19	24–77	13 M; 6 F
			Mean age: 46	
		Group I (Zr): N/D	Group I (Zr): N/D	Group I (Zr): N/D
		Group II (Ti): N/D	Group II (Ti): N/D	Group II (Ti): N/D
Henao et al., 2021 [11]	RCT	30	19–82	14 M; 16 F
		
			Mean age	Group I (Zr): 25%; 75%
		Group I (Zr): 16	Group I (Zr): 54	
		Group II (Ti): 14	Group II (Ti): 56	Group II (Ti): 71.42%; 28.58%
Payer et al., 2015 [18]	RCT	22	23–65	13 M; 9 F
		
		Group I (Zr): N/D	Group I (Zr): N/D	Group I (Zr): N/D
		Group II (Ti): N/D	Group II (Ti): N/D	Group II (Ti): N/D
Koller et al., 2020 [9]	RCT	22	24–77	13 M; 9 F
			Mean age: 46	
		
		Group I (Zr): N/D	Group I (Zr): N/D	Group I (Zr): N/D
		Group II (Ti): N/D	Group II (Ti): N/D	Group II (Ti): N/D
Bienz et al., 2021 [8]	RCT	40	18–80	22 M; 18 F
				55.0%; 45.0%
			Mean age 52.5	
		
		Group I (Zr): 20	Group I (Zr): N/D	Group I (Zr): N/D
		Group II (Ti): 20	Group II (Ti): N/D	Group II (Ti): N/D

Randomized controlled trial (RCT), zirconium (Zr), titanium (Ti), percentage (%), no data (N/D), male (M), female (F).

**Table 3:** Study description.

Author	Follow-Up	Follow-Up Intervals	Type of Implant	Implant Placement Location in Mouth	Implants Number	Outcomes	Surgical Protocol
Siddiqi et al., 2015 [1]	1 y	N/D	ø3.8 × 10 mm ø3.8 × 11.5 mm ø5 × 6 mm ø5 × 10 mm ø5 × 11.5 mm ø5 × 8 mm	Alveolar implants Group I (Zr): 68 Group II (Ti): 60 Median palatal implants: Group I (Zr): 12 Group II (Ti): 10	150 Group I (Zr): 80 Group II (Ti): 70	Plaque index Bleeding index Probing depth Recession Crestal bone level	All the implants were placed according to a flap protocol except the mid-palatal implant. A conventional healing period was followed.
Osman et al., 2014 [12]	1 y	N/D	ø3.8 × 10 mm ø3.8 × 11.5 mm ø5 × 6 mm ø5 × 10 mm ø5 × 11.5 mm ø5 × 8 mm	Region 14: 18 Off-center: 17 Region 24: 18 Mid Palatal: 19 Mid-sympheseal: 19 Region 36: 19 Region 46: 19	129 Group I (Zr): 73 Group II (Ti): 56	Survival Rates Reason for Failure Marginal bone levels	After flap elevation following a mid-crestal incision, drilling sequences were performed according to the instructions of the manufacturer. In all patients, implants were placed no earlier than 6 months after tooth extraction (late implant insertion).
Henao et al., 2021 [11]	1 y	N/D	ø3.3 × 8 mm ø3.3 × 10 mm ø3.3 × 12 mm	Group I (Zr): Central Incisor: 7 Lateral incisor: 8 Canine: 1 Group II (Ti): Central Incisor: 2 Lateral incisor: 10 Canine: 2	30 Group I (Zr): 16 Group II (Ti): 14	Pink esthetic score Probing pocket depth Marginal bone loss Implant crown esthetic index	Incisions were made along the keratinized mucosa of the alveolar ridges with no. 15 BP (Bard-Parker^®^; BD, Franklin Lakes, NJ, USA) surgical blades to reflect full-thickness mucoperiosteal flaps to expose the alveolar bone. Implant sites were flattened, and any sharp areas of the ridge or bone irregularities were removed with wide-diameter carbide burs.
Payer et al., 2015 [16]	2 y	6 m 12 m 18 m 24 m	Group I (Zr): ø4.0 × 10.0 mm ø4.0 × 11.5 mm ø4.0 × 13.0 mm Group II (Ti): ø4.0 × 11.5 mm	Group I (Zr): Max. incisive: 3 Mand. premolar: 1 Mand. molar 12 Group II (Ti): Max incisive: 2 Max molar: 2 Mand premolar: 1 Mand molar: 10	31 Group I (Zr): 16 Group II (Ti): 15	Bleeding on probing; Pink aesthetic score Plaque index Marginal bone level/Bone loss	All implants were placed, without any regenerative procedures involved, no earlier than 6 months after tooth extraction (late insertion protocol). Following a mid-crestal incision, a flap was raised and drills applied as per the manufacturer’s instructions.
Koller et al., 2020 [9]	80 m	30 m 80 m	Group I (Zr): ø4 × 10 mm ø4 × 11.5 mm ø4 × 13 mm; Group II (Ti): ø4 × 11.5 mm	Maxillary implants: 4 Mandibular implants: 24 Premolar region: 2 Incisors region: 5 Molars region: 21	31 Follow-up: 28 Group I (Zr): 14 Group II (Ti): 14	Plaque Index Bleedeing on probing Pink esthetic score Marginal bone loss	After intrasulcular and mid-crestal incisions, full thickness buccal and palatal flaps were raised.
Bienz et al., 2021 [8]	3 m		Group I (Zr): ø4.1 mm Group II (Ti): ø4.1 mm ø4.8 mm	Maxilla Mandible Premolar Molar	80 Group I (Zr): 40 Group II (Ti): 40	Plaque control record Bleeding on probing; Probing depth;	The site was then carefully anesthetized and a mucoperiosteal flap was raised. The flap design consisted of sulcular incisions at the neighboring teeth and a crestal incision, which divided the keratinized tissue equally in the mandible, or connected the palatal line angles in the maxilla. After preparation with a ø 3.5 mm drill, a sealed envelope was opened containing the allocation of the implants according to a computer-generated list.

Zirconium (Zr), titanium (Ti), implant diameter (ø), no date (N/D), months (m), year (y).

**Table 4:** Clinical outcomes of included studies.

Author	Implant Survival (%)	Marginal Bone Loss (mm)	Implant Success (%)	Bleeding on Probing (%)	Plaque Index	Pink Aesthetic Score	Reasons for Failure
Siddiqi et al., 2015 [1]	Group I (Zr): 70.3% Group II (Ti): 77.6.%	Group I (Zr): 0.25 (SD 0.0804) Group II (Ti): 0.132 (SD 0.206)	Group I (Zr): 46/68 (67.6%) Group II (Ti): 40/60 (66.7%)	Group I (Zr): 0.26% (SD 0.36) Group II (Ti): 0.34% (SD 0.42)	Group I (Zr): 0.44 (SD 0.49) Group II (Ti): 0.46 (SD 0.47)	Group I (Zr): N/D Group II (Ti): N/D	Quality of bone type 3. One-piece design of the implant.
Osman et al., 2014 [12]	Group I (Zr): 71.2% Group II (Ti): 82.1%	Group I (Zr): 0.42 (SD 0.40) Group II (Ti): 0.18 (SD 0.47)	Group I (Zr): 57.5% Group II (Ti): 57.1%	Group I (Zr): N/D Group II (Ti): N/D	Group I (Zr): N/D Group II (Ti): N/D	Group I (Zr): N/D Group II (Ti): N/D	Zirconia implants may work better in front tooth replacements than for overdentures.
Henao et al., 2021 [11]	Group I (Zr): 97% Group II (Ti): 100%	Group I (Zr): 0.28 (SD 0.27) Group II (Ti): 0.16 (SD 0.32)	Group I (Zr): 97% Group II (Ti): 100%	Group I (Zr): 0.26% (SD 0.42) Group II (Ti): 0.05% (SD 0.10)	Group I (Zr): 0.01 (SD 0.06) Group II (Ti): 0.01 (SD 0.06)	Group I (Zr): 7.81 (SD 1.72) Group II (Ti): 7.86 (SD 1.29)	N/D
Payer et al., 2015 [18]	Group I (Zr): N/D Group II (Ti): N/D	Group I (Zr): 1.16 (SD 1.01; ME 0.8) Group II (Ti): 0.88 (SD 0.56; ME 0.88)	Group I (Zr):N/D Group II (Ti): N/D	Group I (Zr): 11.9% (SD 9.44; ME 9.5) Group II (Ti): 7.9% (SD 4.98; ME 7.9)	Group I (Zr): 15.88% (SD 6.67; ME 12.0) Group II (Ti): 11.19% (SD 5.69; ME 9.8)	Group I (Zr): 10.33 (SD 2.06; ME 9.0) Group II (Ti): 9.0 (SD 3.54; ME 10.0)	N/D
Koller et al., 2020 [9]	Group I (Zr): 85.7% Group II (Ti): 93.3%	Group I (Zr): 1.51 (SD 0.68; ME1.48) Group II (Ti): 0.92 (SD 0.72; ME 1.03)	Group I (Zr): N/D Group II (Ti): N/D	Group I (Zr): 10.05% (SD 6.43; ME 10.5) Group II (Ti): 15.46% (SD 5.67; ME 16)	Group I (Zr): 11.38 (SD 0.92; ME 11) Group II (Ti): 11.14 (SD 1.07; ME 11)	Group I (Zr): 1.51 (SD 0.68; ME1.48) Group II (Ti): 0.92 (SD 0.72; ME 1.03)	Prosthetic complications, excessive occlusal loading, peri-implant.
Bienz et al., 2021 [8]	Group I (Zr): N/D Group II (Ti): N/D	Group I (Zr): N/D Group II (Ti): N/D	Group I (Zr): N/D Group II (Ti): N/D	Group I (Zr): 66.67% Group II (Ti):50%	Group I (Zr): N/D Group II (Ti): N/D	Group I (Zr): N/D Group II (Ti): N/D	N/D

(Zr) Zirconium, (Ti) titanium, (SD) standard deviation, (ME) means and medians, (%) percentage, (N/D) no date.

**Table 5:** Summary of selected studies for meta-analysis.

Author	Male	Female	Mean Age (Range)	Implants	
				Zr n (%)	Ti n (%)
Siddiqi et al., 2015 [1]	15	4	62 (50–79)	80 (53.3%)	70 (46.7%)
Osman et al., 2014 [12]	15	4	62 (46–80)	73 (56.6%)	56 (43.4%)
Henao et al., 2021 [11]	14	16	55 (19–82)	16 (53.3%)	14 (46.7%)
Payer et al., 2015 [16]	13	9	51 (23–77)	16 (51.6%)	15 (48.4%)

## Data Availability

The original contributions presented in the study are included in the article/Supplementary Materials, further inquiries can be directed to the corresponding author.

## Supplementary Materials

The following supporting information can be downloaded at: https://www.mdpi.com/article/10.3390/jcm13154488/s1, Table S1: Integral table of selected clinical outcomes.
