# Supplementary material for: Comparative Clinical Behavior of Zirconia versus Titanium Dental Implants: A Systematic Review and Meta-Analysis of Randomized Controlled Trials
Source: J Clin Med. 2024 Jul 31;13(15):4488. doi: 10.3390/jcm13154488 (PMC11313197; doi:10.3390/jcm13154488)
Supplement: Supplementary file 1 [file jcm-13-04488-s001.zip › jcm-3118425-supplementary.pdf]

Supplementary Table S1. Integral table of selected clinical outcomes.

AUTHOR	IMPLANT SURVIVAL<br><br>(%)	MARGINAL BONE LOSS	IMPLANT SUCCESS<br><br>(%)	BLEEDING ON PROBING<br><br>(%)	PLAQUE INDEX	PINK AESTHETIC SCORE	REASONS FOR FAILURE
**Siddiqi et Al. [1]**	Group I (Zr): 70.3%<br><br>Group II (Ti):<br><br>77.6%	Group I<br><br>(Zr):<br><br>Baseline<br><br>14:7.70 (1.39)<br><br>11:7.56 (0.92)<br><br>24:7.48 (1.50)<br><br>36:7.20 (1.50)<br><br>31:7.60 (1.59)<br><br>46:7.61 (1.16)<br><br>1 Year<br><br>14:8.06 (1.45)<br><br>11:7.79 (0.88)<br><br>24:7.66 (1.47)<br><br>36:7.63 (1.51)<br><br>31:7.87 (1.74)<br><br>46:7.91 (2.23)<br><br>Change<br><br>14:-0.36(0.32)<br><br>11:-0.23(0.12)<br><br>24:-0.18(0.21)<br><br>36:-0.16(0.26)	Group I (Zr):<br><br>46/68 (67.6%)<br><br>Group II (Ti):<br><br>40/60 (66.7%)	Group I<br><br>(Zr):<br><br>0,26%<br><br>(SD 0,36)<br><br>Group II<br><br>(Ti):<br><br>0,34%<br><br>(SD 0,42)	Group I<br><br>(Zr):<br><br>0,44<br><br>(SD 0,49)<br><br>Group II<br><br>(Ti):<br><br>0,46<br><br>(SD 0,47)	Group I<br><br>(Zr):<br><br>N/D<br><br>Group II<br><br>(Ti):<br><br>N/D	Quality of bone<br><br>type 3.<br><br>One-piece design of the implant.

31:-0.27(0.23)
46:-0.30(0.16)
Group II (Ti):
Baseline
14: 7.08(0.80)
11:7.09 (0.38)
24:7.49 (0.46)
36:7.42 (0.60)
31:7.34 (0.90)
46:7.76 (0.56)
1 year
14:7.19 (0.74)
11:7.35 (0.51)
24:7.49 (0.66)
36:7.49 (0.49)
31:7.39 (0.73)
46:7.84 (0.63)
Change
14:-0.30(0.26)
11:-0.22(0.34)
24:0.07 (0.47)
36:-0.09(0.23)
31:-0.09(0.36)
46:-0.12(0.35)

**Osman**<br>**et Al. [12]**	Group I (Zr):<br>52/73 (71.2%)<br>Group II (Ti):<br>46/56 (82.1%)<br>Mandible:<br>Group I (Zr):<br>30/33 (90.9%)<br>Group II (Ti):<br>23/24 (95.8%)<br>Maxilla:<br>Group I (Zr):<br>22/40 (55.0%)<br>Group II (Ti):<br>23/32 (71.9%)	Group I<br>(Zr):<br>0.42<br>(SD 0.40)<br>Group II<br>(Ti):<br>0.18<br>(SD 0.47)<br>Mandible:<br>Group I<br>(Zr):<br>0.45<br>(SD 0.45)<br>Group II<br>(Ti):<br>0.15<br>(SD 0.47)<br>Group I<br>(Zr):<br>0.38<br>(SD 0.27)<br>Group II<br>(Ti):<br>0.21	Maxilla:	Group I (Zr): 57,5%<br>Group II (Ti):<br>57,1%	Group I<br>(Zr):<br>N/D<br>Group II<br>(Ti):<br>N/D	Group I<br>(Zr):<br>N/D<br>Group II<br>(Ti):<br>N/D	Group I<br>(Zr):<br>N/D<br>Group II<br>(Ti):<br>N/D	Zirconia implants may work better in front tooth replacements than for overdentures.

		(SD 0.48)					
**Henao et Al. [11]**	Group I (Zr):<br>97%<br>Group II (Ti):<br>100%	Group I<br>(Zr):<br>0,28<br>(SD 0,27)<br>Baseline Mean<br>1.86<br>(SD 0.51)<br>95%CI:<br>1.61-2.11<br>12 months Mean<br>2.08<br>(SD 0.55)<br>95%CI:<br>1.78-2.37<br>Group II<br>(Ti):<br>0,16<br>(SD 0,32)<br>Baseline Mean<br>1.78<br>(SD 0.53)<br>95%CI:<br>1.5-2.06	Group I (Zr):<br>97%<br>Group II (Ti):<br>100%	Group I<br>(Zr):<br>0,26%<br>(SD 0,42)<br>Group II<br>(Ti):<br>0,05%<br>(0,10)	Group I<br>(Zr):<br>0,01<br>(SD 0,06)<br>Group II<br>(Ti):<br>0,01<br>(SD 0,06)	Group I<br>(Zr):<br>7,81<br>(SD 1,72)<br>95%CI<br>6.9-8.73<br>Curvature of facial mucosa:<br>10(50.0)<br>Level of facial mucosa:<br>13(81.3)<br>Root convexity/soft tissue color<br>1(6.3)<br>Mesial Papilla:<br>6 (37.5)<br>Distal Papilla:<br>6 (37.5)<br>Esthetic:<br>10(8-10)<br>Speaking:<br>10(9-10)	N/D

12 months Mean<br>1.96<br>(SD 0.48)<br>95%CI:<br>1.68-2.24	Comfort:<br>10.(8-10)<br>Chewing ability:<br>10(8-10)<br>General Satisfaction:<br>10(9-10)<br>Esthetic(mean) 9.00(7-10)<br><br>Group II (Ti):<br>7,86<br>(1,29)<br>95%CI<br>7.11-8.6<br><br>Curvature of facial mucosa:<br>4<br>(28.6)<br>Level of facial mucosa:<br>9<br>(64.3)<br>Root convexity/soft tissue color 1<br>(7.1)

						Mesial Papilla:<br>7(50.0)<br>Distal Papilla:<br>7(50.0)<br>Esthetic:<br>9.5(6-10)<br>Speaking:<br>10(8-10)<br>Comfort:<br>10(8-10)<br>Chewing ability: 10(8-10)<br>General Satisfaction: 10(9-10)<br><br>Esthetic(mean): 8.50(6-10)	
**Payer et Al. [18]**	Group I<br>(Zr):<br>N/D<br>Group II<br>(Ti):<br>N/D	Group I<br>(Zr):<br>Months<br>0: 0.10<br>(SD 0.19;<br>ME 0.0)<br>6: 0.67	Group I<br>(Zr):<br>N/D<br>Group II<br>(Ti):<br>N/D	Group I<br>(Zr):<br>Months<br>0: 10.9%<br>(SD 5.63;<br>ME 11.3)<br>6: 4.8%	Group I<br>(Zr):<br>Months<br>0:15.75%<br>(SD 2.72;<br>ME 15.8)<br>6:14.17%	Group I<br>(Zr):<br>Months<br>0: 6.88<br>(SD 2.1;<br>ME 6.0)<br>6: 8.0	N/D

(SD 0.95;	(SD 0.95;	(SD 6.04;	(SD 3.66;
ME 0.29)	ME 5.3)	ME 15.0)	ME 8.5)
12:1.16	12: 11.9% (SD 9.44;	12:15.88%	12 :10.33
(SD 1.01;	ME 9.5)	(SD 6.67; 7	(SD 2.06;
ME 0.8)	18: 7.6%	ME 12.0)	ME 9)
18:1.2	(SD 6.15;	18:11.9	18: 11.0
(SD 0.76;	ME 9.1)	(SD 4.77;	(SD 2.0;
ME 1.11)	24: 9.1%	ME 18.0)	ME 10)
24:1.48	(SD 4.34;	24:19.38	24: 11.22
(SD 1.05;	ME 9.2)	(SD 0.88;	(SD 1.56;
ME 1.1)		ME 24.0)	ME 11.0)
	Group II		
Group II	(Ti):	Group II	Group II
(Ti):	0: 9.0%	(Ti):	(Ti):
Months	(SD 6.32;	0:11.25%	0: 2.43
0: 0.16	ME 9.0)	(SD 3.67;	(SD 1.27;
(SD 0.24;	6: 11.4%	ME 11.3)	ME 2.0)
ME 0.0)	(SD 0.88;	6:12.88%	6: 6.5
6: 0.4	ME 11.4)	(SD 4.54;	(SD 4.3;
(SD 0.38;	12: 7.9%	ME 12.0)	ME 4.5)
ME 0.34)	(SD 4.98;	12:11.19%	12: 9.0
12: 0.88	ME 7.9)	(SD 5.69;	(SD 3.54;
(SD 0.56;	18: 14.3% (SD 3.89;	ME 9.8)	ME 10)
ME 0.88)	ME 14.3)	18:14.13	18: 8.14
18: 1.15	24: 7.4%	(SD 4.77;	(SD 3.58;

		(SD 0.73;<br>ME 1.12)<br>24: 1.43<br>(SD 0.67;<br>ME 1.1)		(SD 3.39;<br>ME 7.0)	ME 14.2)<br>24:16.05<br>(SD 8.29;<br>ME 16.8)	ME 8.0)<br>24: 10.75<br>(SD 0.71;<br>ME 11)	
**Koller**<br>**et Al. [9]**	Group I (Zr):<br>30 Months<br>85.7%<br>Group II (Ti):<br>30 Months<br>93.3%	Group I<br>(Zr):<br>30 Months<br>1.51<br>(SD 0.68;<br>ME1.48)<br>80 Months<br>1.38<br>(SD 0.81;<br>ME 1.27)<br>Group II (Ti):<br>30 Months<br>0.92<br>(SD 0.72;<br>ME 1.03)<br>80 Months<br>1.17<br>(SD 0.73;<br>ME1.05)	N/D	Group I<br>(Zr):<br>30 Months<br>10.05<br>(SD 6.43;<br>ME10.5)<br>80 Months<br>16.43<br>(SD 6.16;<br>ME18)<br>Group II (Ti):<br>30 Months<br>15.46<br>(SD 5.67;<br>ME 16)<br>80 Months<br>12.60<br>(SD 7.66;<br>ME14.5)	Group I<br>(Zr):<br>30 Months<br>23.68<br>(SD 10.74;<br>ME 23)<br>80 Months<br>11.07<br>(SD 8.11;<br>ME 8.5)<br>Group II<br>(Ti):<br>30 Months<br>21.04<br>(SD 6.09;<br>ME 20.05)<br>80 Months<br>15.20<br>(SD 15.58;	Group I<br>(Zr):<br>30 Months<br>11.38<br>(SD 0.92;<br>ME 11)<br>80 Months<br>11.11<br>(SD 1.27;<br>ME 11)<br>Group II (Ti):<br>30 Months<br>11.14<br>(SD 1.07;<br>ME 11)<br>80 Months<br>11.56<br>(SD 1.01;<br>ME 12)	Prosthetic complications, excessive occlusal loading<br>peri-implant

					ME14)		
**Bienz et Al. [8]**	Group I<br>(Zr):<br>N/D<br>Group II<br>(Ti):<br>N/D	Group I<br>(Zr):<br>N/D<br>Group II<br>(Ti):<br>N/D	Group I<br>(Zr):<br>N/D<br>Group II<br>(Ti):<br>N/D	Group I<br>(Zr):<br>66.67%<br>Group II<br>(Ti):<br>50%	Group I<br>(Zr):<br>N/D<br>Group II<br>(Ti):<br>N/D	Group I<br>(Zr):<br>N/D<br>Group II<br>(Ti):<br>N/D	N/D

(Zr) Zirconium (Ti**)** Titanium (ø) Implant diameter (SD) Mean and standard deviation (ME) Means and medians (ME), (%) Percentage
